# Validation of Short Measures of Work Ability for Research and Employee Surveys

**DOI:** 10.3390/ijerph16183386

**Published:** 2019-09-12

**Authors:** Melanie Ebener, Hans Martin Hasselhorn

**Affiliations:** Department of Occupational Health Science, University of Wuppertal, 42119 Wuppertal, Germany; hasselhorn@uni-wuppertal.de

**Keywords:** work ability, work ability index, WAI, measurement, occupational health, occupational epidemiology

## Abstract

Work ability (WA) is an important concept in occupational health research and for over 30 years assessed worldwide with the Work Ability Index (WAI). In recent years, criticism of the WAI is increasing and alternative instruments are presented. The authors postulate that theoretical and methodological issues need to be considered when developing alternative measures for WA and conclude that a short uni-dimensional measure is needed that avoids conceptual blurring. The aim of this contribution is to validate the short and uni-dimensional WAI components WAI 1 (one item measuring “current WA compared with the lifetime best”) and WAI 2 (two items assessing “WA in relation to the [mental/physical] demands of the job”). Cross-sectional and 12-month follow-up data of two large samples was used to determine construct validity of WAI 1 and WAI 2 and to relate this to respective results with the WAI. Data sources comprise nurses in Europe investigated in the European NEXT-Study (Sample A; N_cross-sectional _= 28,948 and N_Longitudinal _= 9462, respectively) and nursing home employees of the German 3Q-Study (Sample B) where nurses (N = 786; 339, respectively) and non-nursing workers (N = 443; 196, respectively) were included. Concurrent and predictive validity of WAI 1 and WAI 2 were assessed with self-rated general health, burnout and considerations leaving the profession. Spearman rank correlation (ρ) with bootstrapping was applied. In all instances, WAI 1 and WAI 2 correlated moderately, and to a similar degree, with the related constructs. Further, WAI 1 and 2 correlated with WAI moderately to strongly with ρ ranging from 0.72–0.76 (WAI 1) and 0.70–0.78 (WAI 2). Based on the findings and supported by theoretical and methodological considerations, the authors confirm the feasibility of the short measures WAI 1 and WAI 2 for replacing WAI at least in occupational health research and employee surveys.

## 1. Introduction

Work ability is an important concept in occupational health research and practice. Numerous approaches to measure work ability have been developed over the past four decades and there is still dynamic in this field. Responding to increasing criticism we aim to give an overview over the assessment approaches and then discuss theoretical and methodological questions, taking into account new approaches which have been brought up during the last years. Secondly, we investigate the option of using a one-item and a two-item measure for the sound and economic measurement of work ability in large questionnaire studies.

### 1.1. Work Ability—Concept, Theory and Its Historical Development

For more than 30 years the concept of “work ability” has been used in workplace health promotion and work research. In the early 1980s, the Finnish Institute of Occupational Health investigated if occupation-specific pension age limits were justified [1,2]. As part of this research the researchers developed a questionnaire instrument that should allow for predicting which workers would remain longer in working life and which not. This instrument was later called the “Work Ability Index” (WAI). The index should measure “work ability” (WA) in the sense of “How good is the worker at present, in the near future, and how able is he or she to do his or her work with respect to work demands, health, and mental resources?” [2]. Today, the above definition of WA is one of the most widespread definitions of work ability as a recent review of Lederer et al. [3] indicates. WA has shown to predict several outcomes of relevance for occupational health (and further disciplines), e.g., long-term sick leave [4,5,6], premature work-exit [7] and mortality and disability [8].

Concomitantly with developing the instrument, the researchers developed a corresponding conceptual basis, the “Work Ability concept”. With that, the focus shifted from just predicting the time of retirement to monitoring and promoting WA for the sake of prolonging working life [9]. The WA concept has not been static over the time [10], but has been refined over the three decades. Starting point of the WA concept was the insight that work ability is always the result of the interaction of the worker’s resources and his job demands [7], and as such is not a characteristic of the individual worker per se. Later, Ilmarinen and Tuomi [11] described the WA concept with the metaphor “house of work ability”, consisting of four “floors”: (1) health and functional capacities, (2) knowledge and skills, (3) values, attitudes and motivation, and (4) work situation/work demands. This is a very comprehensive approach that has its merits when used as tool for practitioners in occupational health and company consulting. Yet, it can be criticized from a theoretical point of view because it does not make clear how the factors from different “floors” interact with each other and lead to a certain status of WA. Secondly, it is left unclear if the “floors” are seen as *antecedents* of work ability or *parts* of it. It is only recently that researchers tried to measure WA by covering all the “floors” explicitly, namely in the Work Ability Personal Radar [12], implying that the “floors” should be understood as parts of WA.

First in recent years industrial and organisational psychology started to take notice of the WA concept and instrument—decades after the development of the WA concept and WAI and its worldwide use in practice and occupational health research. The time lag is surprising, given the general relevance of the construct in times of an ageing working population. But it has to be kept in mind that firstly, employment participation (and consequently WA as its antecedent) traditionally has not been in the focus of I/O-psychology, and secondly, that the discipline since many years is using related constructs like person-job-fit, employability, subjective ageing or self-efficacy [13]. In the last years, however, WAI and related measures were increasingly used in psychological research (see, e.g., [14]). A description for psychological practitioners was provided three years ago [15] and, recently, Cadiz et al. [13] published a review of the WA literature from an I/O-psychology perspective. However, the latter and also McGonagle et al. [16], who have developed a conceptual model of perceived work ability, criticized a lack of theoretical foundation of the construct WA and tried to integrate it into established psychological theories, requesting more theoretical work on the construct and its measurement.

### 1.2. Measurement of WA by Means of the Work Ability Index—and Its Criticism

There are many questionnaire instruments assessing work ability [13,15]. Probably the first and certainly the most commonly used is the Work Ability Index (WAI, [17]). This is a questionnaire consisting of seven components (we avoid the commonly used term “dimension” because this would imply a scale whose dimensions have been derived from a factor analysis), WAI 1–WAI 7 that—altogether—are meant to constitute individual work ability:

WAI 1 Current work ability compared with the lifetime best (one item);
WAI 2 Work ability in relation to the demands of the job (two items, weighted);
WAI 3 Number of current diseases diagnosed by a physician (original long version: list of 51 diseases, modified short version: list of 14 disease groups [18]);
WAI 4 Estimated work impairment due to disease (one item);
WAI 5 Sick leave during the past year (12 months) (one item);
WAI 6 Own prognosis of work ability two years from now (one item); and
WAI 7 Mental resources (three items).

These components are summed up to a score ranging from 7–49, classified as follows: 7–27 (poor), 28–36 (moderate), 37–43 (good), and 44–49 (excellent) [7]. The cut-off values were derived from the 15th, and 85th percentile of the population in 1981 that has been investigated in the very first WAI study, municipal employees in Finland [7]. Later, the 50th percentile was added, and the resulting cut-offs have been unchanged since that time. 

Considering the history in development of the WAI instrument and the universal relevance of WA, it is not surprising that over time more and more serious concerns with respect to the WAI instrument emerged. These relate to the concept, the cut off values, the design and the content of the questionnaire:

(a) *Conceptual mismatch.* A fundamental critique is that the WAI does not fully cover the comprehensive WA concept (by explicitly inquiring the “four floors”, see above) and that it focusses too much on health aspects, e.g., diagnoses [19]. While this can be understood from the history of development in a field where a classical epidemiological focus on diseases was prevailing and a resource-based view on WA was new [7], this kind of measurement obviously does not mirror the holistic premise of the WA concept.
(b) *Cut off values.* A second major criticism is the continued use of the traditional cut-off values in practice, epidemiology and clinical research, which are merely distribution-based. This does not seem to reflect empirical evidence (even if some researchers have calculated and proposed different WAI cut-off values with respect to specific outcomes, e.g., [20] for predicting the need for rehabilitation). Differences between the four categories low, moderate, good and excellent may just as well be explained by the idea of a continuous variable, which holds richer statistical information than four ordinal categories only [13]. Additionally, the level of work ability in the working population (in Finland) seems to have risen since the times of instrument development [10] and, further, the distribution differs between age groups [21]. Both aspects raise additional questions concerning differentiation and validity of the cut off values of WAI.

The WAI was developed for large epidemiological studies (and was mostly applied as pencil-paper version). Apart from that, the instrument is being used as an individual diagnostic tool for employees, for example applied in interviews within occupational health, it may be part of employee surveys in companies or—finally—it may constitute an interview tool in occupational coaching [22]. The experiences basing on the use of the different modes, however, have led to further criticism of the WAI instrument:

(c) *Length.* The complete WAI is too long for most applications, including large studies that are looking for quite economic measures [19,23,24].
(d) *Privacy.* The use of the WAI has a privacy issue because many employees don´t want to reveal their medical information [18].
(e) *Lack of directivity.* The results of the WAI do not indicate where and how to intervene in case of low scores—both on individual or group levels [25].

### 1.3. New Forms of Measurement of WA

In response to the instruments’ limitations, subsequently, new forms of WA measurement instruments have been developed, most of them directly based on the WAI. On the one hand, the instrument was expanded. Additional aspects and/or antecedents of work ability were included, often primarily for the use in employee surveys. This applies to the ABIplus [26], the Work Ability Survey [27,28] and the Work Ability Personal Radar [12]. For research purposes, these forms of WA assessment may be problematic as with the many additional aspects included (e.g., “social support” in the Work Ability Survey) conceptual overlap with other constructs in a study can hardly be avoided. 

On the other hand, the WAI instrument was reduced. Several short measures for work ability have been developed and used over the years. Most prominent in occupational health research is the Work Ability Score (WAS), which is identical with WAI 1, the single item measuring work ability in relation to lifetime’s best [19]. While it has shown similar relations to sick leave and health-related quality of life [19], it did not identify the risk of disability pension among production workers to the same degree as the WAI [24], nor long-term sickness absence in the Swedish general population [29]. Another solution is the use of WAI 2, the two items covering the ratings of ones work ability in relation to (a) mental and (b) physical work demands. In some instances WAI 2 was used with separated indicators for mental and for physical work ability [30] and sometimes as the complete aspect [14,31]. An analysis of Alavinia et al. [31] showed that of all seven WAI components, WAI 2 had the highest predictive value for disability pension among construction workers. However, knowledge on the validity of WAI 2 is still incomplete.

An advantage of the very short measures WAS and WAI 2 is that they are more easily interpreted than the complete WAI and that they avoid the tilt to health aspects. Cadiz and colleagues [13] criticize that WAI 2 would only capture “mental and physical job demands and does not consider personal and organizational factors” (p. 4). Yet, contrary to that interpretation, it may be assumed that the respondent takes into account any aspect contributing to his personal experience of mental or physical WA. For example, if the respondent cannot concentrate on his tasks due to family problems, he would not rate his mental WA as “excellent”. Mental or physical WA are measures that sum up the personal experience and appraisal of a complex situation, and it is left to the individual how to weigh and combine the aspects that he or she experiences as relevant. This reminds of the perception of and response to the well-established single item question on subjective general health “In general, how do you rate your current health?” which has proven to be a good predictor of future morbidity and mortality [32]. Additionally, McGonagle et al. [16], and very recently Stuer et al. [33], limited their measurement of WA to the general rating of *perceived* work ability, partially with newly developed items. 

In addition to this, there are further short instruments, combining several components of the WAI or simply omitting the delicate WAI 3 (medical diagnoses; e.g., WAI-R [34]), but this does not solve the problem of the health overemphasis in the instrument. 

### 1.4. The WAI is A Formative Measure 

Until today, the most frequent approach to reconsider the WAI measurement was to perform factorial analyses of the WAI components WAI 1–7, assuming the WAI to be a scale. All WAI components loading on a common factor are then supposed to constitute a contextually relevant sub-dimension of WA, at best with a high internal consistency, usually indicated by Cronbach’s alpha.

In several of these validation studies three-factor structures of the WAI components were identified [35,36,37,38]. The focus of scientific methodological discussion, however, lies on two-factorial solutions. When analysing data from large samples of nurses from ten different countries, Radkiewicz et al. [39] found that a two-factorial solution fitted the data best. Martus et al. [40] suggested two correlated factors “subjectively estimated work ability“ and “objective health status“ as an adequate WA model. A recent confirmatory factor analysis by Freyer et al. [41], employing data from a large sample of German employees aged 31–60 years, supported these findings. The authors recommended not to use the one-dimensional WAI sum-score but to compute two sub-scores instead [42]. Cadiz et al. [13], in their overview, took up the notion of two WAI sub-dimensions and sharpened the labelling to “subjective “vs. “objective” work ability. It may be questioned, however, whether a list of own medical diagnoses, generated in a social process, cognitively processed by the individual and later self-reported in a survey may be labelled as “objective”. Apart from the fact, that a self-reported disease list may also be regarded as “subjective”, the notion of “objectivity” might falsely indicate that this measure of WA has a higher validity than “merely perceived” WA of the individual. Further, to equate a list of diseases with WA ([13]: “objective work ability”) does not seem justified: According to an overview given by Varekamp et al. [43] about half of the workers reporting at least one chronic disease do not find their work ability impaired. Thus, a list of diseases may rather be a predictor than a component of work ability. 

Researchers performing factor analysis on the WAI in the attempt to identify sub-dimensions base their operation on the assumption that the WAI was understood and developed under a specific premise: that each of the seven WAI components are indicators of an underlying latent factor “WA”, which causes a substantial covariation among the items. A change in latent WA should consequently lead to a change in all the indicators. However, theoretical considerations on construct measurement brought forward by Fleuren et al. [44] indicate that the WAI is not an example of such *reflective measurement*. 

Instead the WAI may be regarded as a *formative measure*, where a unique constellation of deliberately chosen items constitutes the measure of WA, with the possibility of only low shared variance between the items. If the aspects which are captured by the single items change, WA changes subsequently, but not vice versa. In fact, when constructing the WAI in the 1980s, it seems that item selection was performed as a “method for identifying subjects under the risk at early retirement” [1], a methodological procedure in line with “external construction” [45] (p. 98ff). The result of this procedure was not a scale but an index (Work Ability Index) integrating (a) a subjective global assessment and prognosis of WA (WAI 1, 2, 4 and 6), (b) a selection of potential antecedents (WAI 3 and 5) and, (c) personal resources (WAI 7). Thus, the main purpose of the development of the WAI was not to depict a theory but to predict work- and employment-related outcomes, and a large amount of evidence witnesses that this purpose has been reached very well.

According to Fleuren et al. [44] the misspecification of a formative measurement model as a reflective one “can greatly bias estimates of structural relationships among variables and produce theoretically meaningless indices of model fit”. From our point of view this may, in fact, apply to the many attempts to understand WA better by optimizing its measurement by splitting the WAI instrument into subcomponents, for example by means of factor analysis. 

Yet, if the WAI is a formative measure, as we postulate, this further fuels our question on conceptual mismatch (see above): if every item contributes independently to the measurement of WA, it is even more important that the selection of items sufficiently covers the multitude of influential components that may compose work ability among workers. If WAI 1–WAI 7 show an overemphasis on health and are not covering the theoretically important determinants competence, work situation and also motivation, the measurement will be biased. The fact that several extended WA versions have been developed, such as the Work Ability Personal Radar (WA-PR, [12]) may be indicative of this potential shortcoming. However, the solution cannot be to attempt to fully cover all potentially relevant components of WA in a single questionnaire, a mission deeming virtually impossible. Instead a clear core concept of WA is needed that can be measured parsimoniously.

In summary: WA is a highly relevant concept for occupational health and employment, but from today’s point of view, both conceptualization and measurement exhibit substantial shortcomings. For assessing WA in epidemiological studies and in employee surveys, a uni-dimensional measure is needed that avoids the conceptual blurring of the WAI. Secondly, this measure should avoid privacy issues and be mostly economic. We assume that—among the WAI components—these criteria are fulfilled by the two short measures which rate WA in a generic way, namely WAI 1 and/or WAI 2. While for the validity of WAI 1 some empirical evidence exists, there is a lack of respective evidence concerning WAI 2. Consequently, in this contribution, we investigate the following questions:

- *Question 1:* We will test if WAI 1 and WAI 2, respectively, correlate with constructs conceptually related with work ability, by that following the theoretically-derived nomological network of the constructs. Should this be the case, this contributes to the construct validity of WAI 1 and 2. As correlates we chose
  (a) (self-rated general health, what is a proximal predictor of WA as discussed above (expecting a positive correlation),
  (b) personal burnout, what is known both as predictor and as a consequence of low WA ([46]; expecting a negative correlation),
  (c) and consideration to leave the profession ([47,48]; expecting a negative correlation).
- *Question 2:* We explore the degree to which WAI 1 and WAI 2, each, are comparable with WAI. We do not regard this as the investigation of criterion validity as the value and role of the WAI instrument as criterion remain unclear due to the criticism on the WAI instrument mentioned above. Yet, as the WAI is a well-established instrument in occupational health, we have to investigate and document the relation of the single components WAI 1 and/or WAI 2 with WAI. The comparisons are performed;
  (a) by means of correlations of WAI 1 and WAI 2, each, with WAI, reflecting whether the application of the two short indicators results in the same order of individuals as when the WAI is used; and
  (b) by comparison of the correlations of WAI 1, WAI 2 and WAI, each, with the related constructs mentioned in the paragraph before, indicating whether the short indicators relate to other constructs in a similar way as the WAI.

All questions are investigated cross-sectionally and longitudinally except question 2 (a), where the comparability of the short indicators with WAI at the same time suffices.

## 2. Materials and Methods 

### 2.1. Data

For data analysis, data sets from two large longitudinal written questionnaire studies in the health care sector were used. Both cross sectional as well as longitudinal analyses (12 months apart) were performed. Participants were included in the analyses if they were employed workers for at least ten weekly working hours and had provided valid information for all variables involved in the analyses.

Sample (A) comprises qualified nurses und nursing aids investigated within the European NEXT-Study, a questionnaire study performed from 2002 to 2003 in hospitals, nursing homes and home care services in ten countries. The overall response rates were 55.0% in 2002 and 41.5% in 2003 [49]. For cross-sectional analysis data from 28,948 nurses from ten countries (BE, DE, FIN, FR, IT, N, NL, POL, SLK, UK) were available, for longitudinal analysis data from 9462 nurses from eight countries (not for N, UK). Cross-sectional data from this study have been used before in related analyses of Radkiewicz et al. [39], who followed a different approach.

Sample (B) covers workers in nursing homes which were investigated within the German 3Q-Study. The data used here derives from the first two waves with response rates of 44.0% (2007) and 42.7% (2008) [50]. The sample was split into nurses (n_cross-sectional_ = 786, n_longitudinal_ = 339,) and non-nurses (n_cross-sectional_ = 443, n_longitudinal_ = 196). Non-nurses were predominantly kitchen, administration, housekeeping and laundry staff, and social workers. 

### 2.2. Variables

The Work Ability Index is used as complete score as outlined by Tuomi et al. [51] 1998, yet with the short list of disease groups (14 disease groups instead of 51 diseases) which was shown to replicate the results from the long list with high precision [18]. Over and above, the components WAI 1 and WAI 2 are used as independent variables. WAI 1 consists of a single item “Assume that your work ability at its best has a value of 10 points. How many points would you give your current work ability? (0 = completely unable to work, 10 = Work ability at its best). WAI 2 was assessed by two questions: “How do you rate your current work ability with respect to the physical demands of your work?” and “… mental demands of your work?”, respectively. Response options were: 1, very poor; 2, rather poor; 3, moderate; 4, rather good; 5, very good. The values of the single items were added to a cumulative WAI 2 score with a possible range from 2 to 10. In line with the guidelines [18] the score was not weighted by type of work (physical/mental) because it is assumed that nurses are exposed to both exposures to same degree at work. This was also applied to non-nurses because the dual exposure applies to most of them as well and further to assure comparability of analyses and findings.

*General health* was measured employing the five-item-scale used in the first version of COPSOQ which followed the suggestions of the SF-36 [52,53]. The items to be answered on a five point scale were: ‘in general, would you say your health is’ (answer categories: ‘poor’, ‘fair’, ‘good’, ‘very good’, ‘excellent’), ‘I seem to get sick a little easier than other people’, ‘I am as healthy as anybody I know’, ‘I expect my health to get worse’, ‘my health is excellent’ (answer categories: ‘definitely false’, ‘mostly false’, ‘do not know’, ‘mostly true’, ‘definitely true’). For constructing the scale the original five point scale was set from 1 to 100 following the proposals of the authors [52]. One missing item per participant was tolerated for scale calculation.

*Personal burnout* was assessed using a six-item scale taken from the Copenhagen Burnout Inventory (CBI, [54]). Participants had to indicate on a five-point scale how often they ‘feel tired’, ‘are physically exhausted’, ‘are emotionally exhausted’, ‘think: ‘I can’t take it anymore’, ‘feel worn out’, ‘feel weak and susceptible to illness’. Answer categories were ‘never/almost never’, ‘once or a few times during a month’, ‘once or twice a week’, ‘three to five times during a week’ and ‘(almost) everyday’. We allowed for one missing item when calculating the scale.

*Consideration of leaving the profession* was assessed by one item “How often during the course of the past year have you thought about giving up nursing” with the response options ‘never’, ‘sometimes a year’, ‘sometimes a month’, ‘sometimes a week’, ‘every day’.

### 2.3. Statistical Analyses

As usual, in investigations on construct validation, the relationships of the variables are tested by correlations. Since WAI, WAI 1 and WAI 2 do not to follow a normal distribution [55], [29] and we cannot assume all the indicators to be interval scaled [41], we use the Spearman´s rho (ρ) for ordinal correlation in all analyses. An aspect to be noted is the fact that correlation between WAI 1 and 2, each, with WAI are partial autocorrelations, thus leading to higher coefficients. In cases of reflective measurement, a corrected item-scale correlation would have to be used, excluding the single item from the scale-score before correlating the score with the item. But due to the fact that every item of WAI seems to contribute a quite special information, not reflecting the variance of a single underlying factor (as described above), deleting an item from WAI could possibly mean to change the measure substantially. To avoid this we left the WAI score unchanged. This procedure follows [19]. To assure comparability of the findings, listwise deletion of data was applied in all three samples.

Bootstrapping was used to define 95% confidence intervals of the correlation coefficients. This method is adequate even if a normal distribution of the variable(s) is not given [56]. All the analyses are performed cross-sectionally and longitudinally to enhance the explanatory power of the analyses. We used SPSS Version 25 (IBM Deutschland GmbH, Ehningen, Germany) for our analyses.

## 3. Results

Of the 28,948 participants considered for the cross-sectional NEXT-data analyses, 89.4% were women, the age range was 18–70 years. Among the 9462 nurses selected for NEXT longitudinal analyses, 89.3% were women, the age range was 19–63 years. Of 1498 participants in the 3Q-Study, 1225 met the inclusion criteria for the cross-sectional analyses, 786 nurses and 443 non-nurses (87.5% women, the age range was 18–67 years). A total of 535 participants were included in the 3Q-Study for longitudinal analyses: 339 nurses and 196 non-nurses (86.5% women, age range from 19–65 years).

In sample (A) (nurses in the NEXT-Study), the mean score (all at t1) was 39.4 (SE_Mean_ 0.03) for WAI, 8.1 (0.01) for WAI 1 and 7.6 (0.01) for WAI 2. Among the nurses of sample B (3Q-Study) the respective scores were 39.1 (0.23) for WAI, 7.8 (0.07) for WAI 1 and 7.5 (0.05) for WAI 2. For the non-nursing staff of sample B, all mean scores were higher: 41.3 (0.29) for WAI, 8.2 (0.08) for WAI 1 and 8.1 (0.07) for WAI 2.

### 3.1. Question 1

The correlation coefficients are shown in Table 1 and Table 2. Because all coefficients are significant at a level of α = 0.001, significance levels are not indicated separately in the tables. 

WAI 1 correlates substantially in the expected direction (positively) with general health in all samples in the cross-sectional and longitudinal analyses (rows a, d, g in Table 1 and Table 2, respectively). The correlation of WAI 2 with general health (rows b, e, h) shows the same pattern as it was found for WAI 1. The 95% confidence intervals of the correlation coefficients of WAI 1 and 2 with general health overlap in all instances except in the NEXT cross-sectional sample, where WAI 2 shows a significantly higher correlation with health than WAI 1, yet on low level only (ρ = 0.47 vs. 0.44, rows a and b). The WAI 1 and 2 correlation pattern with burnout follows the pattern described for general health above, although, as expected, in negative direction. Again, WAI 2 shows significantly higher correlation in the NEXT cross-sectional sample (ρ = 0.48 vs. 0.44, rows a and b). Finally, WAI 1 and WAI 2 correlate with considering leaving the profession in the expected direction (negatively), yet at clearly lower levels. Here, no significant differences between the correlations of WAI 1 and 2 were observed in the samples. 

All these results are in line with the supposed nomological network, contributing to the construct validity of each of the two WA indicators. 

### 3.2. Question 2

The first aspect of comparability of WAI 1 and WAI 2 with WAI is their cross-sectional correlation. Table 1 indicates that WAI 1 correlates with WAI positively and substantially in all three samples with ρ ranging from 0.72 to 0.76 and WAI 2 with ρ = 0.70–0.78. Following Ferguson et al. [57] these correlation effects are moderate to strong. In all analyses, ρ of WAI 1 and 2 reach rather similar levels (maximum difference: 0.03) and the 95% CI of WAI 1 and 2 always overlap indicating that none of the two indicators is superior in correlating with WAI. The findings add to the assumption that both, WAI 1 and WAI 2, are closely related measures to the original WAI.

The second aspect of comparability is whether the correlational pattern of WAI 1 and WAI 2 with general health, burnout and consideration to leave the profession is similar to that of WAI. While the substantial correlation between WAI 1 and WAI 2 with the WAI—as indicated above—does suggest that this is the case, it nevertheless needs to be investigated in separate analyses. For this, the three rows of each sample in Table 1 and Table 2 have to be put in relation (e.g., row a, b, c). As expected, it shows that WAI 1, WAI 2 and WAI are always correlated in the same direction with the outcomes general health, burnout and consideration to leave the profession, both in cross-sectional and longitudinal analyses. While the ρ values for WAI and consideration of leaving the profession hardly exceed those of the short indicators, WAI correlates to somewhat higher degree with general health and burnout.

## 4. Discussion

In our analyses, we found that both WAI 1 and WAI 2 correlated clearly and in the expected directions with constructs conceptually related to work ability, that is, self-rated general health, personal burnout and the consideration to leave profession. Furthermore, both short measures correlate substantially with WAI and show the same correlational pattern as WAI with the related constructs. 

Firstly, the construct validity of WAI 1 and WAI 2, as short and clear-cut measures for work ability, was supported by our results: as expected: they correlate with general health, burnout and—to a somewhat lower extent—with consideration to leave the profession. The lower correlation with the latter may be due to fact that this measure was assessed with one (naturally skewed) item only and that the notion of detachment from ones’ profession is a complex phenomenon also strongly influenced by factors beyond WA [58].

Secondly, WAI 1 and 2, each, correlate substantially with WAI, thus ranking individuals widely in a similar order as WAI. That WAI 2 in two instances has significantly higher ρ values than WAI 1 is due to the large sample, the small differences indicate small differences in effect size only, so we cannot see any indication for one of the two short WA indicators correlating systematically stronger with WAI than the other. Yet it should be kept in mind that WAI 2 is measured by two items, thus containing more information. Jääskeläinen et al. [59] suggested that the correlation between WAI and WAI 1 may be high, but not very high, because WAI 1 is relating to the past by assessing current WA in relation to lifetime´s best. Where WA has never been regarded as high by the participants, a low WA could be “best” thus reaching highest scores. In contrast, WAI additionally contains aspects of current and future WA. If this argument was correct, the correlation of WAI 2 (WA in relation to actual work demands) with WAI should be systematically higher than that of WAI 1, yet, we cannot confirm this with our analyses.

Our findings confirms the established practice of using WAI 1 or WAI 2 for measuring WA in questionnaire studies and the respective recommendations given by of other research groups [19,29,59,60,61]. The comparability is further confirmed by the fact that all three WA measures show very similar correlations with general health, burnout and consideration to leave the profession. This is in line with findings of researchers who found similar correlational patterns of WAI and WAI 1 or WAI 2, respectively, with further constructs [19,31,60].

That WAI correlates higher with general health than the short indicators, is not surprising because it contains two explicit health components (WAI 3 and 5) which may inflate the correlation of the constructs through conceptual overlap. According to this view, the correlations of WAI 1 and 2 may be closer to real relationships between work ability and health. This is underlined by results of Lundin et al. [29] who found that WAI correlated with long-term sickness absence stronger than the single components WAI 1 and 2. Inflation of correlation may also be assumed to explain the higher correlations of WAI with burnout as the mental resource component of WAI, WAI 7, has conceptual overlap with this criterion. All in all, the differences in correlation with the outcomes health and burnout between WAI and the short measures are surprisingly low considering that WAI contains 7–8 more items and in addition a long list of diseases. One further aspect to be considered is that the WAI bears a substantially higher risk for non-response than the use of WAI 1 or WAI 2 only, because the large number of items in the WAI, some of them with delicate content, goes along with a higher risk for incomplete response and higher proportion of missings for the sum score. Roelen et al. [24] related the high rate of missings (17%) in their study to the length and complicatedness of the WAI instrument. 

This study was not able to use another conceptually important outcome of WA as criterion, namely *disability pension*. But other studies have investigated this: Alavinia et al. [31] found that each of the components of the WAI had predictive power for future disability pension with WAI 2 revealing the strongest relationship, and Sell [61] found that low WA (measured by an item similar to WAI 1) leads to a higher risk of early labor market exit. Finally, Jääskeläinen et al. [59] showed that WAI 1 like WAI predicted disability pension adequately over a follow-up period of four years in 5251 Finnish municipal employees among women. This supports the idea that WAI 1 and WAI 2 are suitable measures for predicting the timing of the departure from working life, fulfilling the original purpose of the WAI. However, when examining construction workers in the Netherlands, Roelen et al. [24] found that—in contrast to WAI—the discriminatory power of WAI 1 did not suffice to detect individuals with the risk of disability pension, although there was an association between WAI 1 and the outcome. Jääskeläinen et al. [59] found similar results among men over a longer follow-up period and with the outcome taken from register data, labelling the ability of WAS to discriminate men with future disability retirement as “moderate”. This observations—if replicated—may indicate potential for improvement of the short WA measures, possibly towards a more fine grained general measure.

Thus, all in all, based both on theoretical and methodological considerations and on our findings, we confirm the feasibility of the short measures WAI 1 and WAI 2 for replacing WAI and possibly further longer instruments assessing work ability. Below, we discuss this in the light of the established critique on the WAI instrument:

(a) *Conceptual mismatch.* In relation to the full WAI, the components WAI 1 and WAI 2 are clearer in what they measure, namely a general perception of one’s own work ability, thus preventing conceptual blurring. On the one hand, this avoids inflated relations due to conceptual overlap with further constructs in assessments, for example with burnout or health. On the other hand, this makes it unmistakably clear that the WA findings themselves do not identify any of the endless number of specific determinants of WA.
(b) *Cut off values* have been established for WAI 1 [10] (p. 29), but they seem to be chosen only to correspond best with the established WAI categories. Thus, their validation is needed where there is a need for categories. Until today, no cut-off values have been established for WAI 2, which might be future work to be done.
(c) *Length.* The length of WAI 1 and WAI 2 is obviously minimal, contributing to conceptual clarity and probably higher compliance of the respondents. Future studies should analyse whether measures that are conceptually as clear-cut as WAI 1 or 2 but that possibly contain a few more items (e.g., [16,33]) might further improve reliability, validity and distribution characteristics of the WA measurement.
(d) *Privacy issues* are much less a concern with the short indicators than for WAI. This may increase the participants’ compliances and participation rates.
(e) *Lack of directivity* is a need specifically relevant in the field of practical occupational health. The short indicators are even less specific about what has to be done in case of low WA than the WAI. It needs to be discussed if this parsimonious approach is an improvement, giving room for individual interpretation of the measurement and leaving it up to the experts to deal with that information, or if the more global information of a general measure lacks essential important information (e.g., about mental resources). Yet, it may be doubted that it will be possible to capture all—from the point of intervention—relevant determinants of WA in a WA instrument. For the purpose of large studies the advantage of a clear-cut measure seems to outweigh the missing details.

### Strengths and Weaknesses

Among the strengths of the study are the cross validation of results by comparing the findings of three independent study samples. A further strength is the prospective analyses performed for both samples. A methodological weakness might—at first sight—be common method variance. This, however, is unavoidable, because WA, as long as it is understood in a broad way, is a purely subjective concept. Thus, all attempts to capture the components of work ability objectively may be doomed to fail. Yet, when attempting to capture broad concepts summarizing complex and very personal conditions, subjectivity in the assessment may rather be a strength—partly explaining the high predictive power of the measures with respect to objective outcomes (see the discussion of self-rated health as quoted above). A weakness of this study is that it is focusing on the nursing profession, even if it also includes a sample of non-nursing staff in nursing homes. Yet, although our findings exhibit a high consistency across the different samples, they need to be replicated in samples covering further professional groups. 

## 5. Conclusions

Firstly, we confirm that WAI 1 is a suitable measure for WA in epidemiological studies and find that for WAI 2 as well. Secondly, we recommend further work on the measurement of WA, yet, this should be explicitly based on theoretical and methodological considerations as indicated above in this article. Thereby, we suggest to apply a broad view and include all disciplines interested in the measurement of WA, such as epidemiologists, occupational health and psychologists. This might include the consideration of previously ignored well-established constructs closely related to WA (e.g., employability or person-job-fit) and contribute to a research infrastructure of mutual benefit for all disciplines involved. Thirdly, more research should be done on cut-off values of the short WA measures WAI 1 and 2 by relating them to different criteria.

## Figures and Tables

**Table 1:** Cross-sectional analyses—correlation (Spearman’s rho) of WAI 1, WAI 2 and WAI with the outcomes general health, burnout and consideration of leaving nursing. Investigation in three different cross-sectional samples (all t1). The 95% confidence intervals of rho were obtained by bootstrapping. All correlations were significant at *p* < 0.001.

Row			General Health	Burnout	Consideration Leaving Profession	WAI
NEXT-Study: Nurses (n = 28,948)						
a	WAI 1	ρ	*0.44*	*−0.44*	*−0.18*	*0.72*
		*95%CI of* ρ	*0.43 to 0.45*	*−0.45 to −0.43*	*−0.19 to −0.17*	*0.71 to 0.73*
b	WAI 2	ρ	*0.47*	*−0.48*	*−0.19*	*0.70*
		*95%CI of* ρ	*0.46 to 0.48*	*−0.49 to −0.47*	*−0.20 to −0.18*	*0.70 to 0.71*
c	WAI	ρ	*0.58*	*−0.53*	*−0.22*	*1*
		*95%CI of* ρ	*0.57 to 0.59*	*−0.54 to −0.53*	*−0.23 to −0.21*	
3Q-Study: Nurses in nursing homes (n = 786)						
d	WAI 1	ρ	*0.67*	*−0.48*	*−0.32*	*0.76*
		*95%CI of* ρ	*0.62 to 0.71*	*−0.54 to −0.42*	*−0.39 to −0.26*	*0.72 to 0.79*
e	WAI 2	ρ	*0.64*	*−0.56*	*−0.44*	*0.78*
		*95%CI of* ρ	*0.59 to 0.68*	*−0.61 to −0.50*	*−0.50 to −0.38*	*0.75 to 0.81*
f	WAI	ρ	*0.73*	*−0.58*	*−0.44*	*1*
		*95%CI of* ρ	*0.70 to 0.77*	*−0.63 to −0.53*	*−0.50 to −0.38*	
3Q-Study: Non-nurses in nursing homes (n = 443)						
g	WAI 1	ρ	*0.64*	*−0.45*	*−0.34*	*0.75*
		*95%CI of* ρ	*0.57 to 0.70*	*−0.53 to −0.37*	*−0.42 to −0.25*	*0.70 to 0.79*
h	WAI 2	ρ	*0.54*	*−0.50*	*−0.29*	*0.75*
		*95%CI of* ρ	*0.47 to 0.61*	*−0.57 to −0.41*	*−0.37 to −0.19*	*0.70 to 0.79*
i	WAI	ρ	*0.70*	*−0.57*	*−0.34*	*1*
		*95%CI of* ρ	*0.65 to 0.76*	*−0.64 to −0.50*	*−0.42 to −0.27*	

**Table 2:** Longitudinal analyses—correlation (Spearman’s rho) of WAI 1, WAI 2 and WAI with the outcomes general health, burnout and consideration of leaving nursing. Investigation in three different longitudinal samples with all outcomes (t2) being assessed 12 months after t1. The 95% confidence intervals of rho were obtained by bootstrapping. All correlations were significant at *p* < 0.001.

Row			General Health (t2)	Burnout (t2)	Consideration Leaving Profession (t2)	WAI (t2)
NEXT-Study: Nurses (n = 9462)						
a	WAI 1 (t1)	ρ	*0.36*	*−0.34*	*−0.15*	*0.49*
		*95%CI of* ρ	*0.34 to 0.38*	*−0.36 to −0.32*	*−0.17 to −0.13*	*0.47 to 0.51*
b	WAI 2 (t1)	ρ	*0.37*	*−0.36*	*−0.15*	*0.47*
		*95%CI of* ρ	*0.35 to 0.39*	*−0.38 to −0.34*	*−0.17 to −0.13*	*0.45 to 0.49*
c	WAI (t1)	ρ	*0.47*	*−0.43*	*−0.19*	*0.64*
		*95%CI of* ρ	*0.45 to 0.48*	*−0.44 to −0.41*	*−0.21 to −0.17*	*0.62 to 0.65*
3Q-Study: Nurses in nursing homes (n = 339)						
d	WAI 1 (t1)	ρ	*0.49*	*−0.36*	*−0.24*	*0.54*
		*95%CI of* ρ	*0.40 to 0.58*	*−0.46 to −0.27*	*−0.35 to −0.14*	*0.46 to 0.62*
e	WAI 2 (t1)	ρ	*0.51*	*−0.39*	*−0.37*	*0.57*
		*95%CI of* ρ	*0.43 to 0.59*	*−0.48 to −0.30*	*−0.46 to −0.27*	*0.49 to 0.64*
f	WAI (t1)	ρ	*0.62*	*−0.46*	*−0.38*	*0.70*
		*95%CI of* ρ	*0.55 to 0.68*	*−0.54 to −0.37*	*−0.47 to −0.30*	*0.64 to 0.76*
3Q-Study: Non-nurses in nursing homes (n = 196)						
g	WAI 1 (t1)	ρ	*0.46*	*−0.48*	*−0.27*	*0.54*
		*95%CI of* ρ	*0.32 to 0.57*	*−0.59 to −0.35*	*−0.40 to −0.13*	*0.42 to 0.64*
h	WAI 2 (t1)	ρ	*0.47*	*−0.44*	*−0.25*	*0.56*
		*95%CI of* ρ	*0.35 to 0.59*	*−0.56 to −0.32*	*−0.39 to −0.12*	*0.45 to 0.66*
i	WAI (t1)	ρ	*0.50*	*−0.47*	*−0.31*	*0.68*
		*95%CI of* ρ	*0.38 to 0.61*	*−0.58 to −0.36*	*−0.42 to −0.18*	*0.59 to 0.76*
